# Occupational Health Risk Assessment in the Electronics Industry in China Based on the Occupational Classification Method and EPA Model

**DOI:** 10.3390/ijerph15102061

**Published:** 2018-09-20

**Authors:** Ying Cai, Fei Li, Jingdong Zhang, Zixian Wu

**Affiliations:** 1 Research Center for Environment and Health, Zhongnan University of Economics and Law, Wuhan 430073, China; 1993cy@zuel.edu.cn (Y.C.); wuzixianjia@163.com (Z.W.); 2 School of Information and Safety Engineering, Zhongnan University of Economics and Law, Wuhan 430073, China

**Keywords:** occupational health, electronics industry, EPA inhalation risk assessment model, occupational hazards classification, risk management

## Abstract

The awareness of occupational health risk management in the electronics industry is weak in China, and many Chinese occupational health management regulations have not been effectively implemented. China’s current occupational hazards classification method and the Environmental Protection Agency (EPA) inhalation risk assessment model recognized internationally were used to perform health risk assessments for a chip manufacturing company in the electronics industry in order to determine the existing problems and put forward the optimization proposals of the occupational hazards classification method in China. The results showed that the detected concentrations of toxic and harmful chemicals in all testing points did not exceed the occupational health exposure limits in China. According to the EPA inhalation risk assessment model, the highest values of non-carcinogenic risks of ammonia, chlorine, fluoride, sulfuric acid, hydrogen chloride, ethylene glycol, phosphine, boron trifluoride, isopropanol, benzene, and xylene were 5.10, 67.12, 1.71, 45.98, 1.83, 1.43, 160.35, 46.56, 2.52, 5.55, and 5.37, respectively, which means workers in electronic chip manufacturing companies exposed to these chemicals have higher occupational health risks. However, on the basis of the occupational hazards classification method, the occupational health risks of exposure to the toxic and hazardous chemicals are relatively harmless operations. The evaluation results of the EPA inhalation risk assessment model are generally higher than those of the occupational hazards classification method. It’s recommended to refine the value of occupational exposure limit B, taking more characteristics of the hazard factors into account and fuzzifying the parameters to optimize the occupational hazards classification method. At the same time, it is suggested that the electronic chip manufacturing company should conduct anti-virus risk management covering in three aspects: increasing the awareness of occupational hazards, enhancing system ventilation, and improving personal health management measures.

## 1. Introduction

The electronics industry in China is booming. Most people have always believed that the working environment of the electronics industry is relatively clean and safe, without heavy labor and pollution such as smoke and dust. As a result, it has become one of the preferred industries for women’s employment. However, people working in this industry have faced health risks without realizing it, because the common occupational hazards in the electronics industry, such as toxic and hazardous chemicals, extremely small metal dust, radiation, and noise, are almost invisible. Therefore, these conditions can be described as an unpredictable occupational health risk [1]. Over 400 cases of occupational diseases have been newly reported in Guangdong Province every year since the beginning of the 21st century, and more than 30% of these cases are reports of organic solvent poisoning. Organic solvent poisoning has become one of the most serious causes of occupational disease [2], and the electronics industry has been ranked as the first-place industry for organic solvent hazards [3]. In order to protect the safety and health of operators in the electronics industry, China has developed AQ 4201-2008 “Technology Code of Dust and Poison Control for Electronics Industry” [4]. In 2016, QM-J010.0023-2016 “Electronics Factory Environmental Management Code” [5] was newly compiled, standardizing the working environment of electronics enterprises, such as the environmental temperature and humidity, dust particles, air pressure, noise, etc. However, due to the lack of supervision, occupational health risk management in the electronics industry is still in a weak position in China, with occupational health accidents frequently occurring. The US documentary “Who Pays the Price? The Human Cost of Electronics” pointed out that the benzene poisoning situation of employees in the electronics industry is serious in China, by telling the stories of many workers in the electronics industry who are ill due to exposure to toxic and hazardous substances in their jobs. Therefore, while we are proud of the rapid development of the electronics industry, we must also actively respond to the implementation of relevant national occupational health management practices and pay attention to the industry’s hidden environmental and health risk factors, assess its impact on the health of employees, and take related measures. Occupational health risk assessment refers to a series of processes that identify occupational hazards in the workplace, analyze the possible impacts of these occupational hazards on human health, and assess the probability of their occurrence so that corresponding prevention and control measures can be taken to avoid risks [6]. The United States National Academy of Sciences published “The Federal Government’s Risk Assessment: Management Procedures” in 1983, which proposed four basic procedures for risk assessment: hazard identification, dose-response relationships, exposure assessment, and risk characterization [7]. Since then, countries have established health risk assessment methods and corresponding standards based on these four basic procedures, such as the US Environmental Protection Agency (EPA) Risk Assessment Model [8], the International Council on Mining and Metals (ICMM) Health Risk Assessment Module [9], the Australian Occupational Health and Safety Risk Assessment (UQ) Approach [10], the Romanian Occupational Accident and Occupational Disease Risk Assessment (MLSP) Method [11], the Singapore Risk Assessment (MOM) Model [12], and so on. In the 1990s, the foreign risk assessment models began to be introduced into China, with Xie [13] taking the nuclear industry group 222 as an example to discuss the establishment of an occupational health management system in the nuclear field in China for the first time. Since then, China’s occupational health risk assessment has focused on pollution sources and risk sources [14,15]. At present, the major research directions of occupational health risk assessment in China mainly converge on the selection of evaluation models [16,17,18] and exposure studies [19,20] in practical applications. Due to the clean and tidy working environment of the electronics industry, people feel that there is no significant occupational health risk, resulting in few studies on occupational health risk assessment in the electronics industry. The objects of occupational health risk assessment are more concentrated in other sensitive occupations, such as professional pneumoconiosis. Ceballos [21] found that electronics waste recycling may cause various occupational exposures due to the frequent use of manual operations. Chen [22] used different methods to evaluate the occupational health risks of an electronics company’s noise, finding that the noise exposure of the power water treatment plant and the air conditioner room was more serious than that of the press room, the sputtering workshop, and the wastewater blower room, constituting a more serious risk level.

Due to the lack of awareness of occupational health management in the electronics industry and the implementation of the state’s management codes in this area, there are relatively few studies on the assessment of the occupational health risks faced by employees in the electronics industry, which is not conducive to the prevention and treatment of occupational hazards in the electronics industry. China’s current occupational hazards classification method in the workplace and the internationally recognized EPA inhalation risk assessment model are conducted to assess occupational health risks of employees in an electronic chip manufacturing enterprise in China’s electronics industry, in order to compare and analyze the problems existing in the evaluation methods adopted in China, and propose optimization and improvement suggestions. Based on the occupational hazards classification method in the workplace in China and the EPA inhalation risk assessment model, the risk sources that may endanger workers’ health in the workplace of an electronic chip manufacturing company are identified through data collection surveys. Following this, on-site investigations and sampling tests are conducted to obtain the required parameter data for the relevant assessments. Combining the collected relevant toxicological reactions, animal carcinogenicity experiments, and population epidemiological data, we evaluate the occupational environmental health risks to employees caused by long-term work in the working environment of an electronic chip manufacturing company. Based on the evaluation results, the EPA model is used to compare to improve the existing problems in the current occupational hazard operation classification method in China, so as to provide a reference for China’s occupational health risk assessment technology development and occupational health risk management in the electronics industry.

## 2. Materials and Methods

### 2.1. Object and Site Survey

**Figure 1:**
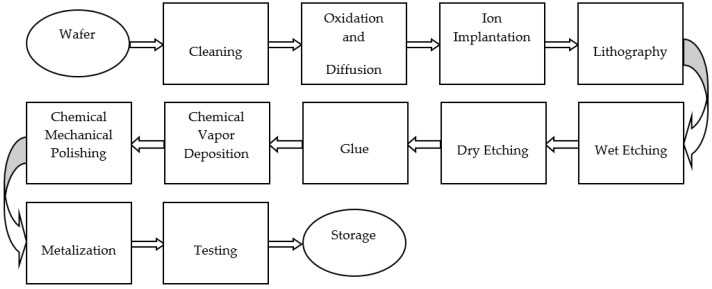
The main technological process of the chip manufacturing company.

The chip manufacturing company belongs to the first batch of key integrated circuit manufacturing companies recognized by the country. With leading chip research and development, production technology and professional production line equipment, the company provides a variety of chip design and manufacturing services. The main technological process and occupational health hazards identification of the chip manufacturing company can be seen in Figure 1 and Table 1.

Due to the constraints of on-site and experimental conditions, the factors of the on-site inspection were mainly chemical hazard factors, including ammonia, chlorine, ozone, fluoride, sulfuric acid, hydrogen chloride, ethylene glycol, phosphine, boron trifluoride, isopropanol, and MACHs.

### 2.2. On-Site Testing and Analysis

According to GBZ 159-2004 “Sampling Practices for Monitoring Harmful Substances in Workplace Air” [23] and GBZ/T 160-2007 “Measurement Methods for Toxic Substances in Workplace Air” [24], each detection factor was tested according to its corresponding 8-h time weighted average allowable concentration (PC-TWA), maximum allowable concentration (MAC) or short-term contact allowable concentration (STEL). We selected representative positions, sampled at different time intervals, and continuously sampled for three working days, which included the period with the highest concentration of harmful substances in the air. The sampling point should be representative and include the highest concentration of working points that the worker has been in contact with for a long time, which was to be the key detection point. The sampler was as close as possible to the worker’s breathing zone without affecting the normal operation of the worker. And the climatic conditions were detected and recorded at the time. The detection methods, basis and other detailed information used in the testing process are shown in Table 2 and Table S1.

### 2.3. Methods

#### 2.3.1. Occupational Hazards Classification Method in the Workplace

The occupational hazards classification method in the workplace is a method of assessing and classifying the risk of occupational diseases for employees in the workplace environment. The basis for the classification of toxic operations includes the weight of three factors: chemical hazards degree, occupational exposure ratio of chemicals, and laborers’ physical labor intensity. The classification index of toxic and hazardous chemicals is calculated using Equation (1) [25]:

(1) $$G=W_{D}\times W_{B}\times W_{L}$$

where G is the grading index. W_D_ is the weight of the chemical hazards degree, and the level of chemical hazards is graded according to GBZ230-2010 “Classification for Hazards of Occupational Exposure to Toxicant” [26]. W_B_ is the weight of the occupational exposure ratio of chemicals, which is according to GBZ 2.1-2007 “Occupational Exposure Limits for Hazards in the Workplace Part 1: Chemical Hazards” [27]. W_L_ is the weight of the laborers’ physical labor intensity, and the level of physical labor intensity is implemented according to GBZ/T 189.10-2007 “Measurement of Physical Agents in Workplace Part 10: Classification of Physical Workload” [28]. The classification results and the values of the weights are shown in Table 3 and Tables S2–S4.

#### 2.3.2. EPA Inhalation Risk Assessment Model

According to the “Personal Health Risk Assessment Manual Part F: Supplementary Guidelines for Inhalation Risk Assessment” [29] issued by the US EPA, the recommended risk assessment method is called the EPA inhalation risk assessment model, which is a kind of assessment method of the health risks of inhaled toxic and harmful substances. The method with two steps in the process of occupational health risk assessment: exposure concentration (EC) estimation and health risk assessment, can be divided into cancer risk assessment and non-carcinogenic risk assessment.

(1) The Estimation of EC

During a certain exposure period, employees may be exposed to the same toxic and hazardous chemicals at different concentrations in several workplaces. The average exposure concentration of toxic and hazardous chemicals at this time is calculated by Equation (2):

(2) $${\mathrm{EC}}_{j}=\underset{i=1}{\sum }\frac{{\mathrm{CA}}_{i}\times {\mathrm{ET}}_{i}\times {\mathrm{EF}}_{i}\times {\mathrm{ED}}_{j}}{{\mathrm{AT}}_{j}}$$

Here, EC_j_ is the average exposure concentration during the j exposure period (mg/m^3^). CA_i_ is the concentration of the toxic and hazardous chemicals in the air of the workplace (g/m^3^). ET_i_ is the exposure time of employees in the i workplace (h/day). EF_i_ is the exposure frequency of employees in the i workplace (day/year). ED_j_ is the duration of exposure during the exposure period (y). AT_j_ is the average exposure time (h), the value of which is ED_j_ × 24 × 365.

(2) Health Risk Assessment

① The non-carcinogenic risk is calculated by Equation (3):

(3) $$\mathrm{HQ}=\frac{\mathrm{EC}}{\mathrm{RfC}}$$

Here, RfC is the inhalation toxicity reference value of the toxicant to be evaluated, also known as the reference concentration (mg/m^3^). HQ is the value of the non-carcinogenic risk. When the value of HQ is greater than or equal to 1, it indicates that the toxic and harmful chemicals have a higher non-carcinogenic risk. Conversely, if the value is lower than 1, it indicates that the toxic and harmful chemicals have a lower non-carcinogenic risk.

② The cancer risk is calculated by Equation (4):

(4) Risk=IUR×EC

In the above equation, Risk is the value of the cancer risk. IUR is the inhalation unit risk (m^3^/mg), also known as the slope coefficient, which refers to the upper estimate value of life-long cancer risk resulting from the continuous exposure of air to a toxic and hazardous chemical concentration of 1 mg/m^3^. When the value of Risk is greater than 10^−6^, it indicates that the toxic and harmful chemicals have a higher cancer risk. Conversely, when it is lower than this value, it indicates that the toxic and harmful chemicals have a lower cancer risk.

The specific values of the two parameters IUR and RfC for the inhalation of toxic and hazardous chemicals can be obtained by consulting the IRIS database on the US EPA website at http://www.epa.gov/iris/index.html.

## 3. Results and Discussion

### 3.1. On-Site Survey and Test Results

According to the on-site investigation, the company had invested significantly in occupational health prevention and control facilities such as anti-dust, anti-heating, and cooling mechanisms, and the layout of the plant, ventilation system, power distribution system, and other designs were reasonable, basically meeting the relevant regulations for occupational health in China. The company conducted occupational health examinations for employees each year, and the rate of inspection was over 90% with no occupational disease patients found. The occupational health hazard factors were sampled and tested at the site for each process of the electronic chip manufacturing company, shown in Table 4 and Tables S5–S15. Occupational exposure limits refer to the level of allowable exposure to harmful factors under which workers can face long-term repeated contact during occupational activities without resulting in harmful effects to the health of the vast majority of those workers [27]. The testing results of all of the on-site sampling points in the company did not exceed the occupational health exposure limits in China.

### 3.2. Risk Assessment Results

It could be seen from the testing results that the on-site sampling values of the toxic and harmful chemicals did not exceed the occupational exposure limits specified by China, resulting in a chemical exposure ratio B of 0. According to Equation (1), for the classification of occupational hazards to toxic and harmful chemicals, regardless of the value of the other parameters, the grading index is 0, which means that the grading index of all chemicals is 0. This indicates relatively harmless operations, as shown in Table 5.

The evaluation results of the EPA inhalation risk assessment method for toxic and hazardous chemicals in the electronic chip manufacturing company are also shown in Table 5. For non-carcinogenic risks, the occupational health risks for employees exposed to chlorine, sulfuric acid, ethylene glycol, phosphine, boron trichloride, isopropanol, benzene, and xylene are higher in all tested posts. There are higher occupational health risks of exposure to ammonia, fluoride, and hydrogen chloride for workers in 76%, 24%, and 9% of the tested posts, respectively. The occupational health risks for ozone and toluene are lower for all employees in the tested posts. For carcinogenic risks, occupational health risk of exposure to benzene is 1.3 × 10^−7^ for workers in the tested posts, indicating that the carcinogenic risk is low.

### 3.3. Comprehensive Analysis of Risk Assessment Results

According to the evaluation results of the occupational hazard factors of the chip manufacturing company, there are great differences in the assessment results of the EPA inhalation risk assessment model and occupational hazards classification method in the workplace. The reasons for these differences were investigated in order to improve the existing problems in the current occupational hazards classification method.

#### 3.3.1. Analysis of the Causes of Differences in Evaluation Results between the Two Methods

That occupational hazards classification method determined the health risk of exposure to toxic and hazardous chemicals in the chip manufacturing company to be relatively harmless. This method was mainly based on the results of the on-site detection of exposure concentrations far below the national occupational exposure limits, resulting in the weight of the occupational exposure ratio to be 0. In this case, regardless of the value of other parameters, the grading index will eventually be 0, determining the classification of relatively harmless operations.

Meanwhile, for the EPA inhalation risk assessment model, because it takes into consideration the high hazards of toxic and hazardous chemicals and the high inhalation unit risk, the health risk of exposure to most toxic and hazardous chemicals in the chip manufacturing company was found to be relatively high. On account of the low RfC, which indicates the high hazard level of a given chemical, even if only the lowest detected concentration of the chemical is used in the calculation, the assessment would still indicate a higher risk for chemicals such as phosphine, ammonia, and chlorine. Therefore, for the assessment of high hazard chemicals, as long as any concentration of such chemicals is detected, the assessment result points to a high level of risk.

#### 3.3.2. Comparison Analysis of the Two Methods

The EPA inhalation risk assessment model has three main advantages. Firstly, the evaluation process involves many parameters, covering a wide range and considering the risk levels of the hazardous factors, the characteristics of acute and chronic effects, and the exposure characteristics of the exposure concentration and exposure time to employees. Furthermore, based on a large number of epidemiological data and experimental results to support the parameters of the reference concentration (RfC) and inhalation unit risk (IUR) values, the assessment results based on field survey results and experimental testing data are ultimately more scientific and objective. Secondly, this model can well assess the carcinogenic and non-carcinogenic effects of chemicals both quantitatively and qualitatively. Thirdly, it is a quantitative assessment method, and thus can obtain a risk value to determine the risk level. There are also two disadvantages to EPA inhalation risk assessment model. On the one hand, this method is only applicable to some chemical toxicants and dusts with risk assessment parameters among the occupational hazards involved in electronic product manufacturing companies, because the corresponding RfCs and IURs of the physical factors and some chemical factors in electronic product manufacturing enterprises are not found in the IRIS database, such as carbon monoxide, sulfur dioxide, nitric oxide, nitrogen dioxide, sodium hydroxide, and so on. On the other hand, the rank of both the cancer risk and the non-carcinogenic risk can only be high level and low level, resulting in the difficulty of subdividing the degree of high risk.

There are three advantages to the occupational hazards classification method in the workplace. First of all, combining the results of field surveys with on-site testing, the evaluation results are comparatively scientific and credible thanks to the relatively objective calculation parameters. Moreover, the method is operable and simple. Secondly, it is suitable for assessing the health risk of various occupational hazards of electronic product manufacturing companies, including toxic and hazardous chemicals, dust, and physical occupational hazards. Last but not least, the grading of the occupational health risk levels is more meticulous, containing four levels of relatively harmless, mild, moderate, and highly hazardous operations, which is of practical significance to help enterprises assess and handle occupational health risks at different levels. The occupational hazards classification method in the workplace also has two disadvantages. One is that the assessment method is relatively incomplete, as it only considers the factors of the degree of harm, the occupational exposure ratio, and the physical strength of laborers. The other is that the values of the parameters used in the calculation are often weighted average concentrations, resulting in the weighted average risk, which may overestimate or underestimate the risk level of occupational hazards in certain positions.

#### 3.3.3. Optimization Suggestions for the Occupational Hazards Classification Method

The existing problems and optimization proposals of the occupational hazards classification method were investigated. Firstly, from the above analysis, since the exposure concentrations of the on-site inspection are far lower than the national occupational exposure limits, the weight of the occupational exposure ratio B is 0, in which case the grading index will eventually be 0, determining the operations to be relatively harmless regardless of the value of other parameters. This result is obviously unreasonable. It is suggested that when the occupational exposure ratio B of a certain hazard factor is less than 1, it is still necessary to assign the weight of B according to the specific size. Furthermore, it is relatively incomplete to only consider the three influencing factors of the degree of hazard, the occupational exposure ratio, and the physical labor intensity of the laborer. Referring to the EPA inhalation risk assessment model, more characteristics of the hazard factors should be taken into account to optimize the parameters, such as the acute and chronic effects as well as the carcinogenic and non-carcinogenic effects of the hazard factors. Last but not least, the parameters used in this assessment method are often the weighted average concentration levels, resulting in a weighted average risk level, which may overestimate or underestimate the risk level of the occupational hazards in certain positions. It is recommended that the parameters used should be fuzzified to obtain the degree of each risk level, i.e., the probability of each risk level.

### 3.4. Risk Management

Combining the on-site detection and assessment results, there are still relatively high occupational health risks in the electronic chip manufacturing company, even if the exposure concentrations of the hazardous and harmful chemicals meet the occupational exposure limit requirements. Therefore, it is recommended that the company should continue to do a good job of occupational hazard prevention and management in accordance with the relevant regulations for the prevention and control of occupational diseases in China. At the same time, the company should strengthen its occupational hazard prevention and control measures in the form of anti-virus action. Above all, each production workshop should identify and determine the types of occupational hazards according to the specific conditions of the workshop, formulate targeted prevention and control measures, and set up occupational hazards warning signs in accordance with the “Marking of Occupational Disease Hazards in the Workplace” in a conspicuous position. Next, it is of great importance to strengthen the ventilation and detoxification facilities in the workshop, especially in the production process positions where toxic and hazardous chemicals exist, such as cleaning, oxidation, diffusion, photolithography, ion implantation, etching, chemical vapor deposition, metal chemical industry ordering, and polishing processes. Ultimately, it is necessary to strictly follow the requirements of pre-job training, strengthen the occupational hazard education of employees, and increase their awareness of the occupational hazards of each post so that they consciously abide by the operating norms and properly wear personal protective equipment.

## 4. Conclusions

According to the EPA inhalation risk assessment model, workers exposed to toxic and hazardous chemicals faced higher occupational health risks in the electronic chip manufacturing company, especially chlorine, sulfuric acid, ethylene glycol, phosphine, boron chloride, ammonia, fluoride, isopropanol, benzene, and xylene. However, on the basis of the occupational hazards classification method, the occupational health risks of exposure to the toxic and hazardous chemicals were relatively harmless, because the on-site testing concentrations of the toxic and hazardous chemicals were found to be lower than the exposure limits. The evaluation results based on the EPA inhalation risk assessment model were generally higher than those based on the occupational hazards classification method. As the on-site testing results met the requirements of occupational exposure limits in China, the assessment results of the occupational hazards classification method were relatively harmless at level 0, while the evaluation results of the EPA inhalation risk assessment model were likely to indicate relatively high risks. It is still necessary to assign the weight of B according to the specific size of the hazard factor when the occupational exposure ratio B of that hazard factor is less than 1. Additionally, more characteristics of the hazard factors should be taken into account to optimize the parameters, such as the acute and chronic effects as well as the carcinogenic and non-carcinogenic effects of the hazard factors. Finally, it is recommended that the parameters used should be fuzzified to obtain the degree of each risk level, in order to solve the problem of uncertainty in occupational health assessment.

## Figures and Tables

**Table 1:** The main technological process and occupational health hazards identification of the chip manufacturing company.

Main Process	Possible Chemical Hazards
Cleaning	Hydrogen peroxide, sulfuric acid, phosphoric acid, nitric acid, hydrofluoric acid, hydrochloric acid, isopropyl alcohol, acetone. and ammonia
Oxidation and Diffusion	Diborane and phosphine
Ion Implantation	Phosphine, arsine, and boron trifluoride
Lithography	TMAH, cyclopentanone, isopropanol, ethylene glycol, and butyl acetate
Wet Etching	Sulfuric acid, hydrofluoric acid, nitric acid, phosphoric acid, ammonia, and hydrogen peroxide
Dry Etching	Sulphur hexafluoride, hydrogen chloride, and chlorine
Chemical Vapor Deposition	Hydrogen chloride, fluoride, silane, phosphine, arsine, diborane, chlorine trifluoride, ammonia, chlorine, and nitrogen oxides
Chemical Mechanical Polishing	Ammonia, hydrofluoric acid, hydrogen peroxide, and potassium hydroxide
Metalization	Copper, ammonia, hydrofluoric acid, sulfuric acid, nitric acid, fluoride, and hydrogen peroxide
Public Auxiliary Facilities	Hydrochloric acid, sulfuric acid, sodium hydroxide, ammonia, fluoride, hydrogen fluoride, chlorine, isopropyl alcohol, acetone, ozone, carbon monoxide, carbon dioxide, methane and MACHs

**Table 2:** On-site detection factors, basis, methods, instruments, and model.

No.	Detection Factors	Detection Basis	Detection Methods	Detection Instrument
1	Ammonia	GBZ/T160.29-2004	Nanoreagent spectrophotometry	JH722S visible spectrophotometer
2	Chlorine	GBZ/T160.37-2004	Methyl orange spectrophotometry	JH722S visible spectrophotometer
3	Ozone	GBZ/T160.32-2004	Eugenol spectrophotometry	JH722S visible spectrophotometer
4	Fluoride	GBZ/T160.36-2004	Ion selective electrode method	PHS-3D pH meter
5	Sulfuric acid	GBZ/T 160.33-2004	Barium chloride turbidimetry	JH722S visible spectrophotometer
6	Hydrogen chloride	GBZ/T160.37-2004	Mercury thiocyanate spectrophotometry	JH722S visible spectrophotometer
7	Ethylene glycol	GBZ/T160.48-2007	Gas chromatography	Fl 9790II gas chromatograph
8	Phosphine	GBZ/T160.30-2004	Ammonium molybdate spectrophotometry	JH722S visible spectrophotometer
9	Boron trifluoride	GBZ/T160.27-2004	Phenylglycolic acid spectrophotometry	JH722S visible spectrophotometer
10	Isopropanol	GBZ/T160.48-2007	Gas chromatography	Agilent 7890 A gas chromatograph
11	MACHs	GBZ/T160.42-2007	Gas chromatography	Agilent 7890 A gas chromatograph

**Table 3:** The operation classification results of toxic and harmful chemicals.

Grading Index Range	Level
0	Level 0 (relatively harmless operations)
0 < G ≤ 6	Level I (mildly hazard operations)
6 < G ≤ 24	Level II (moderately hazardous operations)
G > 24	Level III (highly hazardous operations)

**Table 4:** Test results of occupational health hazard factors in the electronic chip manufacturing company.

Hazard Factors	Concentration Range (mg/m^3^)	Exposure Limits (mg/m^3^)	Exposure Time (h)	Judgement Result
Ammonia	0.500–7.600	20	10	Qualified
Chlorine	0.030–0.030	1	10	Qualified
Ozone	0.060–0.170	0.3	10	Qualified
Fluoride	0.010–0.036	2	10	Qualified
Sulfuric acid	0.013–0.411	1	10	Qualified
Hydrogen chloride	0.011–0.109	0.75	10	Qualified
Ethylene glycol	1.700–1.700	20	10	Qualified
Phosphine	0.090–0.143	0.3	10	Qualified
Boron trifluoride	1.213–1.803	3	10	Qualified
Isopropanol	1.500–1.500	350	10	Qualified
Benzene	1.200–1.200	6	10	Qualified
Toluene	0.900–0.900	50	10	Qualified
Xylene	1.600–1.600	50	10	Qualified

**Table 5:** Evaluation results of two methods of occupational hazards classification for the electronic chip manufacturing company.

Hazard Factors	EPA Inhalation Risk Assessment Model			Occupational Hazard Classification
	HQ	High Risk Ratio	Low Risk Ratio	Risk Level
Ammonia	0.34–5.10	76%	24%	Level 0 relatively harmless operation
Chlorine	67.12	100%	0%	Level 0 relatively harmless operation
Ozone	0.05–0.14	0%	100%	Level 0 relatively harmless operation
Fluoride	0.48–1.71	24%	76%	Level 0 relatively harmless operation
Sulfuric acid	1.45–45.98	100%	0%	Level 0 relatively harmless operation
Hydrogen chloride	0.18–1.83	9%	91%	Level 0 relatively harmless operation
Ethylene glycol	1.43	100%	0%	Level 0 relatively harmless operation
Phosphine	100.68–160.35	100%	0%	Level 0 relatively harmless operation
Boron trifluoride	31.32–46.56	100%	0%	Level 0 relatively harmless operation
Isopropanol	2.52	100%	0%	Level 0 relatively harmless operation
Benzene	5.55	100%	0%	Level 0 relatively harmless operation
	(Risk: 1.3 × 10^−7^)	0%	100%	
Toluene	0.06	0%	100%	Level 0 relatively harmless operation
Xylene	5.37	100%	0%	Level 0 relatively harmless operation

## Supplementary Materials

The following are available online at http://www.mdpi.com/1660-4601/15/10/2061/s1, Table S1: The detailed information for sampling and measuring; Table S2: Classification and value of chemical hazards; Table S3: Classification and value of the ratio of the occupational exposure to chemical; Table S4: Classification and value of physical strength; Table S5: The on-site testing result of ammonia; Table S6: The on-site testing result of chlorine; Table S7: The on-site testing result of ozone; Table S8: The on-site testing result of fluoride; Table S9: The on-site testing result of sulfuric acid; Table S10: The on-site testing result of hydrogen chloride; Table S11: The on-site testing result of ethylene glycol; Table S12: The on-site testing result of phosphine; Table S13: The on-site testing result of boron trifluoride; Table S14: The on-site testing result of isopropanol; Table S15: The on-site testing result of MACHs.
