# Supplementary material for: Occupational Health Risk Assessment in the Electronics Industry in China Based on the Occupational Classification Method and EPA Model
Source: Int J Environ Res Public Health. 2018 Sep 20;15(10):2061. doi: 10.3390/ijerph15102061 (PMC6210263; doi:10.3390/ijerph15102061)
Supplement: Supplementary file 1 [file ijerph-15-02061-s001.pdf]

Table S1. The detailed information for sampling and measuring.

No.	Detection Factors	Sampling devices	Sampling medium	Set-up conditions	Detection limits	Measuring duration	Sampling number
1	Ammonia	Large bubble absorber	0.5 mol/L sulfuric acid	420 nm wavelength	0.2 μg/ml	0.5 L/min; 15 min	102
2	Chlorine	Large bubble absorber	Absorbent [a]	515 nm wavelength	0.2 μg/ml	0.5 L/min; 10 min	33
3	Ozone	Large bubble absorber	Eugenol	560 nm wavelength	0.06 μg/ml	2 L/min; 15 min	60
4	Fluoride	Impregnated filter paper	Dipping solution [b]	——	0.06 μg/ml	5 L/min; 15 min	147
5	Sulfuric acid	Sampling clip	Microporous membrane	420 nm wavelength	1 μg/ml	5 L/min; 15 min	48
6	Hydrogen chloride	Porous glass plate absorber	4 g/L sodium hydroxide solution	460 nm wavelength	0.4 μg/ml	0.5 L/min; 15 min	63
7	Ethylene glycol	Silicone tube	200 mg/100 mg silica gel; 2% isopropyl alcohol solution (desorption)	FID; Column 2 m × 4 mm, FFAP：Chromosorb WAW = 10 : 100; column temperature 170℃; vaporization chamber temperature 290℃; detection chamber temperature 220℃; nitrogen flow 40 ml/min	100 μg/ml	0.5 L/min; 15 min	21
8	Phosphine	Porous glass plate absorber	0.1 mol/L potassium permanganate solution: 1 mol/L sulfuric acid solution = 1 : 1 (*v*/*v*)	680 nm wavelength	0.1 μg/ml	5 L/min; 15 min	18
9	Boron trifluoride	Porous glass plate absorber; Sampling clip	8 g/L sodium hydroxide solution; glass fiber filter	633 nm wavelength	0.25 μg/ml	1 L/min; 15 min	9
10	Isopropanol	Activated carbon tube	100 mg/50 mg activated carbon; 1% isobutanol carbon disulfide solution (desorption)	FID; Column 2 m × 4 mm, FFAP：Chromosorb WAW = 10 : 100; column temperature 90℃; vaporization chamber temperature 200℃; detection	0.4 μg/ml	0.1 L/min; 15 min	96

				chamber temperature 220℃; nitrogen flow 40 ml/min			
11	MACHs	Activated carbon tube	100 mg/50 mg activated carbon; carbon disulfide (desorption)	FID; Column 30 m × 0.53 mm × 0.2 μm, FFAP; column temperature 80℃; vaporization chamber temperature 150℃; detection chamber temperature 150℃; nitrogen flow 40 ml/min	Benzene 0.9 μg/ml; toluene 1.8 μg/ml; xylene 4.9 μg/ml	0.1 L/min; 15 min	9

[a] 0.1000 g of methyl orange was weighed to dissolve in about 100 ml of 40~50°C water. After cooling 20ml of 95% (*v/v*) ethanol was added to be transferred to 1000 ml volumetric flask, and water was added to the mark. 1 ml of this solution is equivalent to approximately 24 μg of chlorine. Then about 50 ml of this solution, equivalent to 1.25 mg of chlorine, was taken in a 500 ml volumetric flask, 1 g of potassium bromide was added, and water was added to the mark. 1 ml of this solution corresponds to 2.5 μg of chlorine. Further, 400 ml of this solution was mixed with 100 ml of a sulfuric acid solution (2.57 mol/L) to obtain an absorption liquid; [b] 8 g of sodium hydroxide was dissolved with water, and 20 ml of glycerol was added, then water was added to dilute to 1 L.

Table S2. Classification and value of chemical hazards.

Chemical Hazard Classification	Weight (W_D)
Mild hazard	1
Moderate hazard	2
Severe hazard	4
Extreme hazard	8

Table S3. Classification and value of the ratio of the occupational exposure to chemical

Occupational Exposure Ratio	Weight (W_B)
$B \le 1$	0
$B > 1$	B

Table S4. Classification and value of physical strength

Physical Labor Intensity Classification	Weight (W_L)
I (Mild)	1.0
II (Moderate)	1.5
III (Severe)	2.0
IV (Extreme)	2.5

Table S5. The on-site testing result of ammonia

Sampling Points	C1 (mg/m^3)	C2 (mg/m^3)	C3 (mg/m^3)	Average (mg/m^3)	Exposure Time (h)
PWNIS01	1.9	1.7	1.6	1.733	10
PWHSG01	1.7	1.5	0.6	1.267	10
PDALD62	2.4	2	1.6	2.000	10
PWRMT02	1.7	1.8	1.8	1.767	10
PTOXA02	1.7	2.2	1.9	1.933	10
PTBPA01	1.9	2	1.8	1.900	10
PWOXE01	2	2.1	4.1	2.733	10
PWGOC01	1.5	1.8	1.9	1.733	10
PWRCA02	1.9	1.7	1.8	1.800	10
PWWPR61	1.7	2.1	1.6	1.800	10
PDLSN02	1.8	2	1.9	1.900	10
PWRPL01	1.9	1.7	1.8	1.800	10
PTSCA21	2.4	1	2.7	2.033	10
PTDCN21	2	1	1.7	1.567	10
PTTTN61	2.1	2.1	2.3	2.167	10
PTCBA01	1.7	1.8	0.5	1.333	10
PTHDA01	0.5	1.2	1.8	1.167	10
PDTSN01	0.5	0.5	1.2	0.733	10
PWNPS01	1.7	3.1	2	2.267	10
PWPRS05	1.5	1.7	2.8	2.000	10
PWNIS61	3	2.4	2.1	2.500	10
PWPRS02	1.5	1.8	2.2	1.833	10
PTSNA22	0.5	0.5	0.5	0.500	10
PTSCN21	1.1	0.7	0.5	0.767	10
PTBDA21	0.5	0.5	0.5	0.500	10
PTNIA01	0.5	0.5	2.2	1.067	10
PTHAC61	3.1	2.3	1.5	2.300	10
PDSIN04	3.5	1.3	2.6	2.467	10
PDSIN12	4	1.6	4.2	3.267	10
OSIF FA Lab Chemical Hood 6QHOD01	3.4	2.1	2.2	2.567	10

Flammable gas room	4.9	5.8	5.4	5.367	10
Waste collection room	17.3	3.1	2.4	7.600	10
Chemical room	2.6	2.5	5.4	3.500	10
OS1F Chemical Hood 6QHOD01	5.4	3.8	3.5	4.233	10

Table S6. The on-site testing result of chlorine

**Sampling Points**	**C1 (mg/m³)**	**C2 (mg/m³)**	**C3 (mg/m³)**	**Average (mg/m³)**	**Exposure Time (h)**
PEMEL22	0.03	0.03	0.03	0.030	10
PEMEL24	0.03	0.03	0.03	0.030	10
PEVTO71	0.03	0.03	0.03	0.030	10
PEPLA52	0.03	0.03	0.03	0.030	10
PESDP61	0.03	0.03	0.03	0.030	10
PEPLL05	0.03	0.03	0.03	0.030	10
PEPLL02	0.03	0.03	0.03	0.030	10
PEITS01	0.03	0.03	0.03	0.030	10
PEWTN61	0.03	0.03	0.03	0.030	10
PDAPY61	0.03	0.03	0.03	0.030	10
Corrosive gas room	0.03	0.03	0.03	0.030	10

Table S7. The on-site testing result of ozone

**Sampling Points**	**C1 (mg/m³)**	**C2 (mg/m³)**	**C3 (mg/m³)**	**Average (mg/m³)**	**Exposure Time (h)**
PWWPR61	0.06	0.06	0.06	0.060	10
PWHSG01	0.06	0.06	0.06	0.060	10
PWGOC01	0.06	0.06	0.06	0.060	10
PWRCA02	0.06	0.06	0.06	0.060	10
PTTEA21	0.21	0.21	0.06	0.160	10
PTBDA21	0.23	0.21	0.07	0.170	10
PTHAC61	0.06	0.06	0.2	0.107	10
PTHPA01	0.06	0.06	0.16	0.093	10
PTBPA01	0.08	0.08	0.12	0.093	10
PTTEA02	0.06	0.06	0.06	0.060	10
PTOXA02	0.06	0.06	0.06	0.060	10
PWRMT02	0.06	0.06	0.06	0.060	10
PTALD01	0.06	0.07	0.07	0.067	10
PDHFO01	0.17	0.07	0.2	0.147	10
PTSCA21	0.09	0.1	0.06	0.083	10
6CTMD02	0.07	0.06	0.06	0.063	10
PTTES91	0.06	0.07	0.08	0.070	10
PDMAO01	0.06	0.06	0.08	0.067	10
PTSNA22	0.06	0.08	0.15	0.097	10
6CGRD01	0.09	0.08	0.07	0.080	10

Table S8. The on-site testing result of fluoride

Sampling Points	C1 (mg/m³)	C2 (mg/m³)	C3 (mg/m³)	Average (mg/m³)	Exposure Time (h)
PEMEL 24	0.012	0.014	0.013	0.013	10
PEMEL 22	0.012	0.013	0.016	0.014	10
PEOXA 21	0.014	0.014	0.012	0.013	10
PEWTN 61	0.01	0.01	0.013	0.011	10
PECHG 61	0.013	0.01	0.01	0.011	10
PENSA 01	0.011	0.01	0.01	0.010	10
PECTA 01	0.01	0.01	0.01	0.010	10
PEOXT 02	0.011	0.02	0.016	0.016	10
PQFIB 01	0.01	0.01	0.01	0.010	10
PEOXM 91	0.012	0.014	0.012	0.013	10
PEPLA 52	0.011	0.011	0.012	0.011	10
PECHM 61	0.012	0.013	0.014	0.013	10
PEITS 01	0.011	0.011	0.012	0.011	10
PEOXA 91	0.01	0.01	0.01	0.010	10
PECHG 91	0.012	0.012	0.014	0.013	10
PEBEL 01	0.012	0.012	0.014	0.013	10
PEVTO 71	0.01	0.01	0.01	0.010	10
PTTEA 21	0.01	0.01	0.011	0.010	10
PEDTI 21	0.01	0.01	0.01	0.010	10
PTBOA 21	0.011	0.012	0.014	0.012	10
PTDCW 21	0.012	0.011	0.01	0.011	10
PTSNA 22	0.01	0.012	0.014	0.012	10
PTNIA 01	0.012	0.011	0.011	0.011	10
PTWPN 01	0.012	0.014	0.014	0.013	10
PTTEA 02	0.013	0.012	0.011	0.012	10
PTOXA 02	0.01	0.01	0.013	0.011	10
PTHDA 01	0.014	0.012	0.011	0.012	10
PTLBA 01	0.012	0.01	0.01	0.011	10
PTTTN 61	0.011	0.011	0.01	0.011	10
PTBPA 01	0.01	0.01	0.012	0.011	10
PTSTN 01	0.01	0.013	0.013	0.012	10
PTPGA 01	0.015	0.01	0.011	0.012	10
PESDP 61	0.012	0.01	0.01	0.011	10
PEPLL 02	0.027	0.035	0.031	0.031	10
PTALD 01	0.026	0.027	0.033	0.029	10
PTTES 91	0.037	0.021	0.029	0.029	10
PTSCA 21	0.033	0.027	0.029	0.030	10
PEOXL 21	0.021	0.027	0.029	0.026	10
PEPLL 05	0.02	0.02	0.023	0.021	10
PTHAC 61	0.021	0.02	0.016	0.019	10
PTHPA 01	0.023	0.021	0.02	0.021	10
PEOXL 24	0.019	0.018	0.023	0.020	10
S1F FA Lab 6QIB02	0.018	0.017	0.017	0.017	10
OS1F FA Lab Cylinder rack	0.017	0.015	0.014	0.015	10

OS1F chemical Lab solvent Hood	0.015	0.016	0.015	0.015	10
Flammable gas room	0.035	0.031	0.033	0.033	10
Corrosive gas room	0.035	0.033	0.037	0.035	10
Toxic gas room	0.037	0.035	0.035	0.036	10
Inert gas room	0.037	0.026	0.042	0.035	10

Table S9. The on-site testing result of sulfuric acid.

Sampling Points	C1 (mg/m³)	C2 (mg/m³)	C3 (mg/m³)	Average (mg/m³)	Exposure Time (h)
PPRTC01	0.013	0.013	0.013	0.013	10
PTECP71	0.383	0.058	0.013	0.151	10
PTEPN21	0.013	0.422	0.701	0.379	10
PWPWC71	0.193	0.504	0.536	0.411	10
PWSIE02	0.985	0.013	0.076	0.358	10
PWNPS01	0.013	0.013	0.013	0.013	10
PWPRS02	0.013	0.013	0.013	0.013	10
PWPRS05	0.013	0.013	0.013	0.013	10
AMT	0.013	0.013	0.013	0.013	10
Wastewater treatment room	0.013	0.013	0.013	0.013	10
Acid and alkali waste collection chamber	0.013	0.013	0.013	0.013	10
Wastewater Pharmacy	0.013	0.013	0.013	0.013	10
Chemical room	0.387	0.013	0.013	0.138	10
FA lab 6QHOD01	0.697	0.013	0.013	0.241	10
Acid and alkali waste gas treatment	0.013	0.013	0.013	0.013	10
OS 1F Chemical Lab Chemical Hood 6QHOD01	0.013	0.013	0.013	0.013	10

Table S10. The on-site testing result of hydrogen chloride.

Sampling Points	C1 (mg/m³)	C2 (mg/m³)	C3 (mg/m³)	Average (mg/m³)	Exposure Time (h)
PDGOX02	0.011	0.011	0.011	0.011	10
PDHOX05	0.011	0.011	0.011	0.011	10
PDPOX03	0.011	0.011	0.011	0.011	10
PWRPL01	0.011	0.011	0.305	0.109	10
PWWPR61	0.011	0.011	0.011	0.011	10
PWCOS01	0.135	0.043	0.036	0.071	10
PWGOC01	0.011	0.011	0.011	0.011	10
PWRCA02	0.011	0.011	0.011	0.011	10
PWNIS01	0.011	0.011	0.011	0.011	10
PWOXE01	0.011	0.011	0.011	0.011	10
PWNIS61	0.011	0.011	0.011	0.011	10
PWNPS01	0.011	0.011	0.011	0.011	10
PWPRS02	0.011	0.011	0.011	0.011	10
PWPRS05	0.011	0.011	0.011	0.011	10

Pure water treatment room acid tank operation	0.011	0.011	0.011	0.011	10
Wastewater pharmacy	0.011	0.011	0.011	0.011	10
Chemical room	0.011	0.011	0.011	0.011	10
Chemical lab solvent Hood	0.011	0.011	0.011	0.011	10
FA lab 6QHOD01	0.011	0.011	0.011	0.011	10
Corrosive gas room	0.011	0.011	0.011	0.011	10
OS 1F Chemical Lab Chemical Hood 6QHOD01	0.011	0.011	0.011	0.011	10

Table S11. The on-site testing result of ethylene glycol.

**Sampling Points**	**C1 (mg/m^3)**	**C2 (mg/m^3)**	**C3 (mg/m^3)**	**Average (mg/m^3)**	**Exposure Time (h)**
PEOXL 21	1.7	1.7	1.7	1.700	10
PEOXL 24	1.7	1.7	1.7	1.700	10
PEOXA 21	1.7	1.7	1.7	1.700	10
PEPLA 52	1.7	1.7	1.7	1.700	10
PESDP 61	1.7	1.7	1.7	1.700	10
PECHG 61	1.7	1.7	1.7	1.700	10
PENSA 01	1.7	1.7	1.7	1.700	10

Table S12. The on-site testing result of phosphine.

**Sampling Points**	**C1 (mg/m^3)**	**C2 (mg/m^3)**	**C3 (mg/m^3)**	**Average (mg/m^3)**	**Exposure Time (h)**
PIHEI 01	0.07	0.04	0.16	0.090	10
PIMCI 02	0.2	0.06	0.04	0.100	10
PIHCI 03	0.04	0.31	0.04	0.130	10
PDUPY 03	0.21	0.07	0.15	0.143	10
PTHDA 01	0.04	0.08	0.19	0.103	10
PTPGA 01	0.2	0.09	0.04	0.110	10

Table S13. The on-site testing result of boron trifluoride.

**Sampling Points**	**C1 (mg/m^3)**	**C2 (mg/m^3)**	**C3 (mg/m^3)**	**Average (mg/m^3)**	**Exposure Time (h)**
PIHEI01	1.4	2.99	0.93	1.773	10
PIHEI03	1.4	2.95	1.06	1.803	10
PIHEI02	0.12	1.38	2.14	1.213	10

Table S14. The on-site testing result of isopropanol.

**Sampling Points**	**C1 (mg/m^3)**	**C2 (mg/m^3)**	**C3 (mg/m^3)**	**Average (mg/m^3)**	**Exposure Time (h)**
PWPRS 05	1.5	1.5	1.5	1.500	10
PWPRS 02	1.5	1.5	1.5	1.500	10
PWWTE 61	1.5	1.5	1.5	1.500	10

PWNIS 61	1.5	1.5	1.5	1.500	10
PTBDA 21	1.5	1.5	1.5	1.500	10
PTTEA 21	1.5	1.5	1.5	1.500	10
PTSNA 22	1.5	1.5	1.5	1.500	10
PTSCA 21	1.5	1.5	1.5	1.500	10
PTHAC 61	1.5	1.5	1.5	1.500	10
PTALD 01	1.5	1.5	1.5	1.500	10
PCOXA 02	1.5	1.5	1.5	1.500	10
PCOXA 04	1.5	1.5	1.5	1.500	10
PWRPL 01	1.5	1.5	1.5	1.500	10
PWNPS 01	1.5	1.5	1.5	1.500	10
PTHPA 01	1.5	1.5	1.5	1.500	10
PCCUA 21	1.5	1.5	1.5	1.500	10
PCCUA 23	1.5	1.5	1.5	1.500	10
PTTEA 02	1.5	1.5	1.5	1.500	10
PTOXA 02	1.5	1.5	1.5	1.500	10
PTBPA 01	1.5	1.5	1.5	1.500	10
PTTES 91	1.5	1.5	1.5	1.500	10
PEOTX 02	1.5	1.5	1.5	1.500	10
PWWPR 61	1.5	1.5	1.5	1.500	10
PEITS 01	1.5	1.5	1.5	1.500	10
PWGOC 01	1.5	1.5	1.5	1.500	10
PWNIS 01	1.5	1.5	1.5	1.500	10
PWOOS 01	1.5	1.5	1.5	1.500	10
PWOXE01	1.5	1.5	1.5	1.500	10
OS1F FA Lab 6QHOD 02	1.5	1.5	1.5	1.500	10
Solvent room	1.5	1.5	1.5	1.500	10
Organic solvent waste collection chamber	1.5	1.5	1.5	1.500	10
Chemical Lab Chemical Hood 6QHOD 01	1.5	1.5	1.5	1.500	10

Table S15. The on-site testing result of MACHs.

MACHs	Sampling Points	C1 (mg/m^3)	C2 (mg/m^3)	C3 (mg/m^3)	Average (mg/m^3)	Exposure Time (h)
Benzene	OS 1F Chemical Lab GC-MS 6QGC01	1.2	1.2	1.2	1.200	10
Toluene	OS 1F Chemical Lab GC-MS 6QGC01	0.9	0.9	0.9	0.900	10
Xylene	OS 1F Chemical Lab GC-MS 6QGC01	1.6	1.6	1.6	1.600	10
